# Interphase Cytogenetic Analysis of Micronucleated and Multinucleated Cells Supports the Premature Chromosome Condensation Hypothesis as the Mechanistic Origin of Chromothripsis

**DOI:** 10.3390/cancers11081123

**Published:** 2019-08-06

**Authors:** Antonio Pantelias, Ioanna Karachristou, Alexandros G. Georgakilas, Georgia I. Terzoudi

**Affiliations:** 1 Laboratory of Health Physics, Radiobiology & Cytogenetics, Institute of Nuclear & Radiological Sciences & Technology, Energy & Safety, National Centre for Scientific Research “Demokritos”, 15341 Agia Paraskevi, Greece; 2 DNA Damage Laboratory, Physics Department, School of Mathematical and Physical Sciences, National Technical University of Athens (NTUA), 15780 Zografou, Greece

**Keywords:** premature chromosome condensation (PCC), micronuclei, chromothripsis, chromosome shattering, chromosomal instability, RO-3306, PCC hypothesis, PCC dynamics

## Abstract

The discovery of chromothripsis in cancer genomes challenges the long-standing concept of carcinogenesis as the result of progressive genetic events. Despite recent advances in describing chromothripsis, its mechanistic origin remains elusive. The prevailing conception is that it arises from a massive accumulation of fragmented DNA inside micronuclei (MN), whose defective nuclear envelope ruptures or leads to aberrant DNA replication, before main nuclei enter mitosis. An alternative hypothesis is that the premature chromosome condensation (PCC) dynamics in asynchronous micronucleated cells underlie chromosome shattering in a single catastrophic event, a hallmark of chromothripsis. Specifically, when main nuclei enter mitosis, premature chromatin condensation provokes the shattering of chromosomes entrapped inside MN, if they are still undergoing DNA replication. To test this hypothesis, the agent RO-3306, a selective ATP-competitive inhibitor of CDK1 that promotes cell cycle arrest at the G2/M boundary, was used in this study to control the degree of cell cycle asynchrony between main nuclei and MN. By delaying the entrance of main nuclei into mitosis, additional time was allowed for the completion of DNA replication and duplication of chromosomes inside MN. We performed interphase cytogenetic analysis using asynchronous micronucleated cells generated by exposure of human lymphocytes to γ-rays, and heterophasic multinucleated Chinese hamster ovary (CHO) cells generated by cell fusion procedures. Our results demonstrate that the PCC dynamics during asynchronous mitosis in micronucleated or multinucleated cells are an important determinant of chromosome shattering and may underlie the mechanistic origin of chromothripsis.

## 1. Introduction

First insights into the central role of chromosomes in cancer development emerged in Boveri’s hypothesis, formulated more than 100 years ago, which posited that somatic genetic changes leading to uncontrolled cell proliferation caused cancers. By examining cancer cells under the microscope, Boveri observed the presence of peculiar chromosomes and, essentially, proposed that cancers are abnormal clones of cells characterized and caused by abnormalities of hereditary material [1]. At present, one consistent hallmark of human cancer genomes is chromosomal instability [2] and the formation of numerical and structural alterations in chromosomes, including deletions, duplications, inversions, and translocations [3,4,5,6,7,8,9]. Potential causes leading to chromosome instability include spindle assembly defects, chromosome segregation defects, erroneous repair of DNA damage, telomere dysfunction, and DNA replication stress. For example, it has been shown that deficiencies in key DNA repair factors for homologous recombination (HR) or canonical non-homologous end joining (cNHEJ) in a p53 deficient environment can result in frequent chromosomal catastrophic events. These complex genome rearrangements can stimulate tumor development by the amplification of oncogenes, for instance [10,11]. These and other pivotal regulatory cellular function defects are also currently considered as the driving mechanisms for the initiation of the striking phenomenon termed chromothripsis, on the basis of its chromosomal hallmarks that point to an underlying process involving chromosome (chromo) shattering (thripsis) [12]. Chromothripsis is characterized by extensive genomic rearrangements with a large number of breakpoints but a limited number of oscillating copy number states [12,13,14,15]. The study of the molecular mechanism that leads to chromothripsis remains a challenging research topic [16].

Chromothripsis has been extensively studied in primary tumors of diverse histological origin, and recent studies suggest that it may be far more common that initially inferred from low resolution DNA copy number data, with a frequency of more than 50% in several cancer types [17]. In 2013, Korbel and Campbell [14] suggested criteria to discriminate between rearrangements resulting from chromothripsis and from stepwise DNA alterations. However, chromothripsis is not specific to cancer, as similar random joining of chromosomal fragments has been observed as well in the germline [18]. Despite the progress achieved in elucidating this phenomenon, a detailed picture of the complete process of its mechanistic origin remains elusive, while much remains to be discovered regarding its prevalence and consequences [13,19,20]. Several non-mutually exclusive hypotheses have been proposed and discussed in recent reviews [21,22,23,24]: Fragmentation and subsequent reassembly of a single lagging chromosome encapsulated in aberrant extranuclear chromatin bodies surrounded by a nuclear envelope called micronuclei (MN) [13,25,26,27,28,29]; fragmentation of dicentric chromosomes and induction of chromothripsis through the breakage-fusion-bridge cycle following telomere crisis [30,31]; chromosomal shattering and reassembly through excessive shattering of telomeric DNA by ionizing radiation (IR) during cell mitosis [32], when chromosomes are condensed and individualized; induction of chromosomal rearrangements through fragmentation of chromatin by abortive apoptosis; and chromosome shattering and reassembly through premature chromosome condensation (PCC) within micronucleated cells and asynchronous cell-cycle progression between main nucleus and MN [13,25,26,33].

A hallmark of chromothripsis is chromosome shattering, and the precise timing of this event is crucial for determining the underlying mechanisms. Does the massive accumulation of fragmented DNA that could drive chromothripsis originate, through MN disruption and aberrant DNA replication inside MN, before main nuclei enter mitosis? Or does it arise as a consequence of the PCC dynamics in asynchronous micronucleated cells when main nuclei enter mitosis? Consistent with the latter case, we have previously proposed that chromatin condensation, triggered by the PCC process when main nuclei enter mitosis, causes the collapse of replication forks and the massive accumulation of fragmented DNA in MN [26]. Nevertheless, it is important to note that both potential origins of shattering might play a significant role. Additionally, the molecular events underlying the fragility of replication forks in mitosis and their subsequent collapse remain incompletely understood [19,34]. Following exposure to various genotoxic agents such as IR, MN can encapsulate acentric fragments of chromosomes, as well as whole anaphase lagging chromosomes. Interestingly, even though MN maintain several characteristics of main nuclei, studies differ regarding their ability to undergo normal DNA replication and transcription, exhibit normal DNA damage response, and assemble normal nuclear envelopes [27,35,36]. 

In the present study, we conducted interphase cytogenetic analysis of asynchronous micronucleated human lymphocytes and Chinese hamster ovary (CHO) multinucleated cells, to investigate whether the dynamics of the PCC process are an important determinant of the mechanism that leads to chromothripsis. Our hypothesis, based on earlier work [26,37,38,39], is that the chromatin condensation activated by the PCC process exerts mechanical stress on chromosome sites in MN that are still undergoing normal DNA replication when main nuclei enter mitosis. As a result, the DNA replication forks in MN may collapse and eventually convert into double strand breaks (DSBs), thus causing chromosome shattering in a single catastrophic event. Hence, the extent of chromosome shattering should be proportional to the number of MN replication forks at the entry of main nuclei into mitosis.

In order to test the above hypothesis, we applied IR to generate asynchronous micronucleated cells and employed cell fusion procedures in exponentially growing cells to obtain asynchronous multinucleated cells. Subsequently, using the agent RO-3306, a selective ATP-competitive inhibitor of CDK1 that promotes cell cycle arrest at the G2/M boundary, we controlled and delayed the entrance of main nuclei into mitosis. The rationale is that this delay defers the initiation of the PCC process and allows time for MN to complete DNA replication and progress towards G2 phase. This strategy enabled to test at the cytogenetic level whether MN disruption or defective DNA replication in MN is necessary for chromosome shattering to occur. In fact, if disruption or aberrant DNA replication takes place in MN before the entry of main nuclei into mitosis, one envisions that the duplication of chromosomal material residing in MN is expected to be markedly impaired and/or shattered even if the agent RO-3306 is applied. However, according to our hypothesis, chromosome shattering is not expected if complete DNA replication takes place in MN before the entry of main nuclei into mitosis. Indeed, we demonstrate here, for the first time, that anaphase lagging chromosomes entrapped in MN can proceed into G2 phase without any shattering or impairment in their duplication. Altogether, our data demonstrate that an important determinant of chromosome shattering is the asynchrony between MN and main nuclei, supporting thus the PCC hypothesis as the mechanistic basis of chromothripsis initiation.

## 2. Results

Through four sets of experiments, we demonstrate that the dynamics of the PCC process in asynchronous micronucleated or multinucleated cells can be the cause of shattering of chromosome segments, which are entrapped in MN or heterophasic nuclei, and are still undergoing DNA replication when main nuclei enter mitosis. Specifically, a critical determinant of the extent of chromosome shattering is the degree of cell cycle asynchrony between MN and main nuclei. Furthermore, we show that chromosomes entrapped in MN, if given sufficient time, can undergo chromatid disjunction and complete normal DNA replication, without any impairment in their duplication. This is revealed by means of a thorough G2-PCC assessment upon the entry of main nuclei into mitosis, and the interphase cytogenetic evidence obtained here supports the PCC hypothesis as the mechanistic origin of chromothripsis.

### 2.1. In Asynchronous Micronucleated Cells Generated by γ-Irradiation of G_0_-lymphocytes, the PCC Process Triggers Shattering of Chromosomes in MN Still Undergoing DNA Replication When Main Nuclei Enter Mitosis

**Figure 1:**
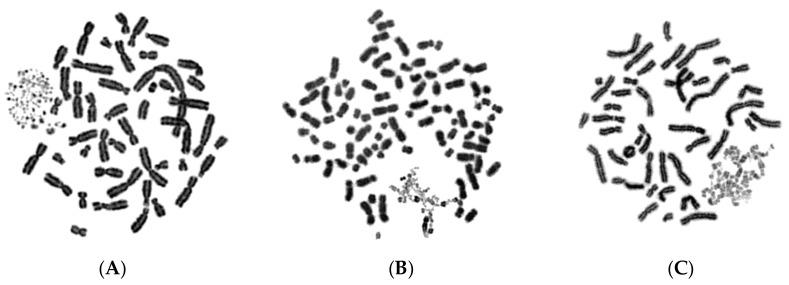
Early-S, mid-S and late-S MN PCCs. In micronucleated cells generated by exposure of G_0_-lymphocytes to ionizing radiation (IR) (4 Gy), chromosome shattering of chromosomal material entrapped in micronuclei (MN) can occur through premature chromosome condensation (PCC), if MN are in S phase when main nuclei enter mitosis. Upon entry of main nuclei into mitosis, the nuclear envelope of MN disassembles and, through the mitotic cyclin B1-CDK1 activity and histone phosphorylation, the shattering and morphology of prematurely condensed chromosomes (PCCs) characterizes the stage in S phase of MN. Based on the degree of completion of DNA replication, the MN PCCs can be classified as: (**A**) early-S, (**B**) mid-S, and (**C**) late-S phase. Darkly stained metaphasic chromosomes belong to main nuclei, while lightly stained shattered chromosomal material indicate anaphase lagging chromosomes entrapped in MN.

In the first set of experiments, asynchronous micronucleated cells were generated by in vitro γ-irradiation (4 Gy) of G_0_-human blood lymphocytes to induce chromosomal aberrations and anaphase lagging chromosomes, which will be entrapped by nuclear envelopes and form micronuclei, as described in Materials and Methods. The effect of the mechanical stress exerted by the dynamics of PCC on chromosomes trapped in MN when main nuclei enter mitosis was thoroughly examined. Even though the ultimate fate of chromosomes within MN in asynchronous micronucleated cells remains unclear, these experiments enable the visualization of the chromosomes and their progression through the different stages of the cell cycle. Upon entry of main nuclei into mitosis, the nuclear envelope of MN disassembles and, through the activity of mitotic cyclin B1-CDK1 and histone phosphorylation, chromatin condensation occurs. The cell cycle phase of MN can be inferred through the observation of the chromatin architecture and morphology of the prematurely condensed chromosomes. Therefore, based on the degree of completion of DNA replication, the chromosomes in the interphase MN can be classified as being in G1, early-S, mid-S, late-S, or G2 phase. The typical appearance of chromosome shattering in S phase MN, classified as early-S, mid-S, and late-S phase PCCs in interphase MN, is shown in Figure 1.

**Figure 2:**
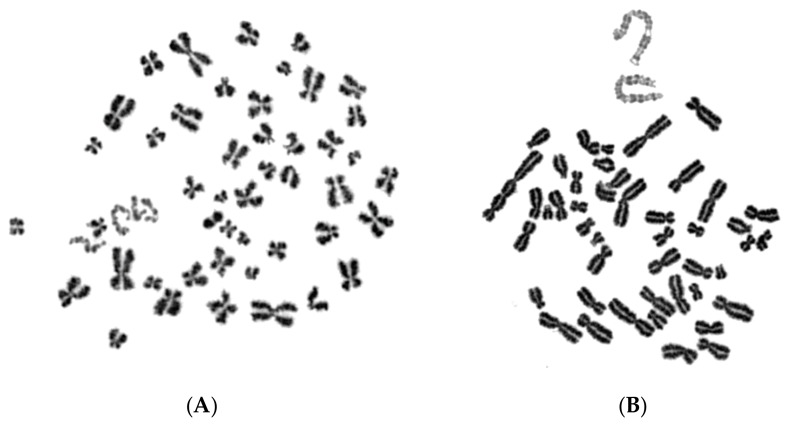
G1 and G2 MN PCCs. When main nuclei in heterophasic micronucleated cells generated by exposure of G_0_-lymphocytes to IR (4 Gy) enter mitosis, chromosome shattering is not observed in the MN PCCs if the morphology of chromosomes entrapped in MN is classified as G1 (**A**) or G2 phase (**B**). Anaphase lagging chromosomes entrapped in MN can undergo chromatid disjunction (**A**), and complete DNA replication without impairment in their duplication (**B**). The different level of chromatin condensation between MN chromosomes and those of the main nuclei leads to dissimilar shades of staining, making them easily distinguishable. Darkly stained metaphasic chromosomes belong to main nuclei, while lightly stained chromosomes were entrapped in MN.

Figure 2 presents the typical appearance of G1 and G2 phase PCCs in interphase MN. The cytogenetic assessment of the chromosome preparations show that PCC-induced chromosome shattering is only observed in MN that are still undergoing DNA replication when main nuclei enter mitosis. Indeed, chromosome shattering was never detected when the chromosomes in MN were in G1 phase or in G2 phase.

**Figure 3:**
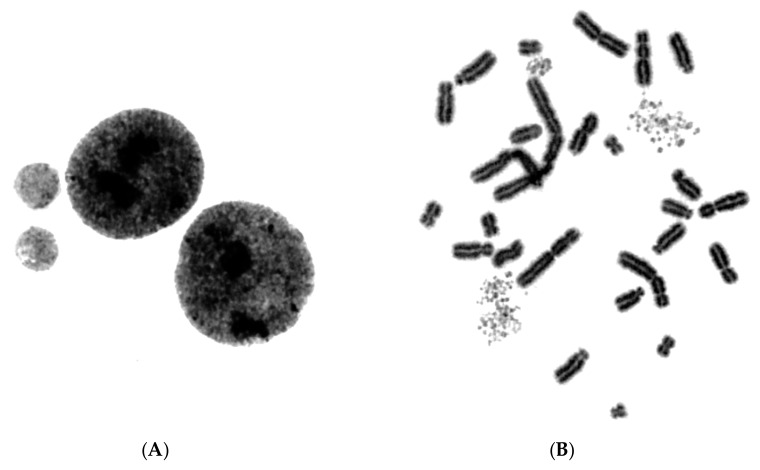
MN PCCs are visualized only when main nuclei enter mitosis. Through rigorous cytogenetic assessment, more than a thousand heterophasic micronucleated cells per experimental point were analyzed for nuclear envelope rupture and shattering of chromosomal material entrapped in MN. (**A**) Chromosome shattering in MN was never observed if main nuclei were not in mitosis. (**B**) Shattering of chromosomal material entrapped in MN was solely detected if, and only if, the main nuclei were in M phase and the MN were in S phase.

**Figure 4:**
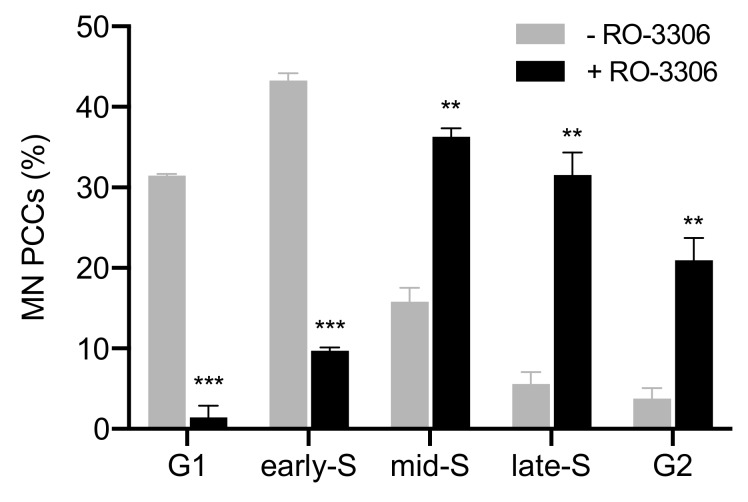
Cell cycle distribution of MN PCCs generated by irradiated G_0_-lymphocytes. Frequencies of five different cell cycle phase categories of MN PCCs scored in heterophasic micronucleated cells, generated by irradiation of G_0_-lymphocytes, upon entry of main nuclei into mitosis. Based on the progress of completion of DNA replication, the chromosomes in the interphase MN can be classified as being in G1, early-S, mid-S, late-S, or G2 phase. The agent RO-3306, a selective ATP-competitive inhibitor of CDK1, was used for 20 h to delay the entrance of main nuclei into mitosis, thus allowing time for completion of DNA replication in MN. In the absence of RO-3306, 75% of MN PCCs are in G1 and early-S phase, while only 25% are in mid-S, late-S, and G2 phase. In the presence of RO-3306, only 11% of MN PCCs are in G1 and early-S phase, while 36% are in mid-S, 32% in late-S, and 21% in G2. As the DNA replication progresses and MN proceed towards G2 phase, the observed chromosome shattering decreases. (Mean ± SEM based on two independent experiments; a total of 457 MN PCC spreads were analyzed; ** *p* ≤ 0.01, *** *p* ≤ 0.001).

Moreover, more than a thousand micronucleated cells per experimental point were assessed for chromosome shattering in asynchronous micronucleated cells. Chromosome shattering was visualized in MN only when main nuclei were in M phase, as shown in Figure 3. The percentages of cells in the five different categories of MN PCCs obtained under these experimental conditions are presented in Figure 4 (grey). In order to delay the entrance of main nuclei into mitosis, the agent RO-3306, a selective ATP-competitive inhibitor of CDK1, was applied to asynchronous heterophasic micronucleated cells for 20 h. Subsequently, the RO-3306 agent was washed out and the main nuclei were allowed to proceed to mitosis and then blocked in metaphase, using Colcemid for 4 h. The corresponding percentages of cells obtained for the five different categories of induced PCCs in MN are also presented in Figure 4 (black). The results of this set of experiments show that, in the absence of RO-3306, most of the MN PCCs are in G1 and early-S phase, whereas in the presence of this agent, most of MN PCCs are in mid-S, late-S, and G2 phase.

**Figure 5:**
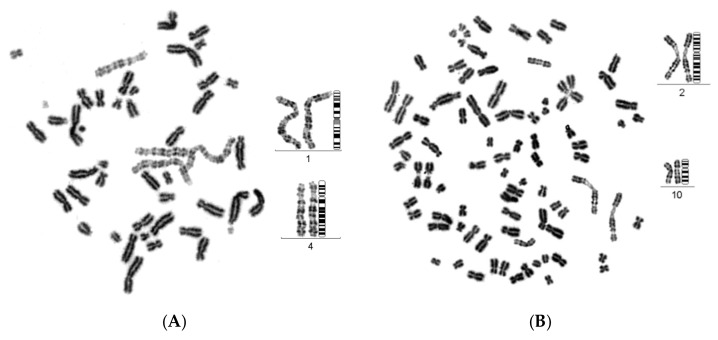
Normal DNA replication can take place in MN. Following radiation exposure of human lymphocytes to generate micronucleated cells, anaphase lagging chromosomes entrapped in MN can undergo chromatid disjunction and complete DNA replication, without any shattering or impairment in their duplication, if entrance of main nuclei into mitosis is sufficiently delayed by RO-3306. (**A**) Duplication of chromosomes 1, 4 and a fragment entrapped in MN. (**B**) Duplication of chromosomes 2 and 10 entrapped in MN. Based on G-banding ideograms for chromosomes 1, 4, 2, and 10, there is no impairment in the duplication of these chromosomes entrapped in MN.

In order to test whether chromosome shattering, which is a hallmark of chromothripsis, arises in MN through the PCC process and not as a result of a massive accumulation of fragmented DNA inside MN occurring before main nuclei enter mitosis, we exploited the unique features of RO-3306. Indeed, the presence of the RO-3306 agent delayed the entry of main nuclei into mitosis, thus allowing the progression of DNA replication in the chromosomes entrapped in MN. As a result, an increased number of MN completed DNA replication and chromosome duplication, and thus entered G2 phase without any chromosome shattering. Following aberrant mitosis, if given sufficient time, anaphase lagging chromosomes entrapped in MN can undergo chromatid disjunction and complete normal DNA replication, without any impairment in their duplication or chromosome shattering, as revealed through G2-PCC assessment upon the entry of main nuclei into mitosis. Figure 5A shows complete replication and normal duplication of chromosomes 1, 4 and a fragment entrapped in MN, whereas in Figure 5B, the duplication of chromosomes 2 and 10 without any shattering is presented.

**Figure 6:**
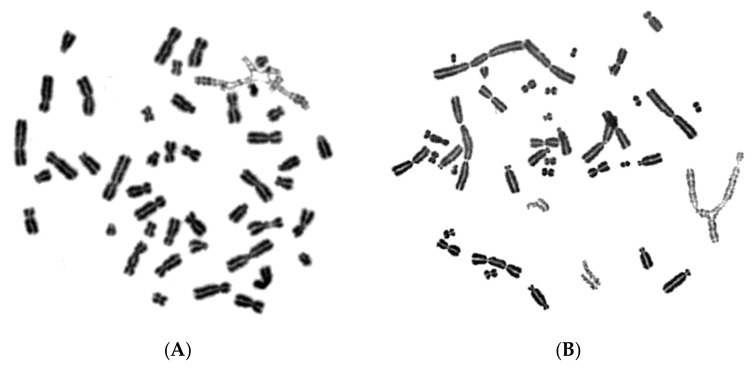
Aberrant chromosomes entrapped in MN can be duplicated. If entrance of main nuclei into mitosis is sufficiently delayed by RO-3306, radiation-induced aberrant anaphase lagging chromosomes entrapped in MN can also undergo chromatid disjunction and complete DNA replication, without any apparent chromosome shattering. (**A**) Duplication of aberrant chromosome 1 (lightly stained) entrapped in MN following radiation exposure (4 Gy) of human G_0_-lymphocytes. (**B**) Duplication of aberrant anaphase lagging chromosomes (lightly stained) entrapped in MN following 4 Gy exposure of G_0_-lymphocytes.

Figure 6 shows replication and duplication of aberrant chromosomal material entrapped in MN following radiation exposure, again without any apparent chromosome shattering.

If massive accumulation of fragmented DNA inside MN had occurred before main nuclei entered mitosis, the duplication of the chromosomes in Figure 5 and Figure 6 residing in MN would be markedly impaired and result into their shattering, which is not observed in the above experiments. Therefore, these results suggest that chromothripsis may not arise from a massive accumulation of fragmented DNA and unrepaired DSBs inside MN before main nuclei enter mitosis.

### 2.2. The PCC Process Underlies the Mechanistic Origin of Chromosome Shattering by a One-Step Cellular Catastrophic Event in Asynchronous Micronucleated Cells Generated by γ-Irradiation of Lymphocytes in the G1/S Phase Border

**Figure 7:**
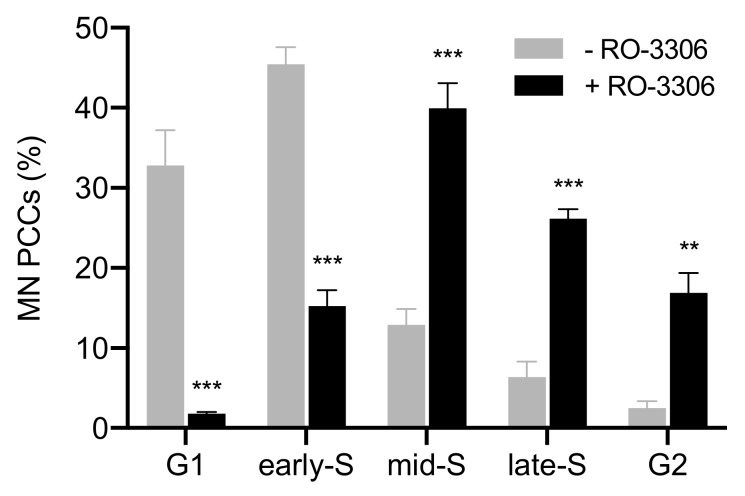
Cell cycle distribution of MN PCCs generated by irradiated G1/S lymphocytes. Frequencies of five different cell cycle phase categories of MN PCCs scored in heterophasic micronucleated cells, generated by irradiation of PHA-stimulated lymphocytes at the highly radiosensitive G1/S border to induce an increased yield of MN. In the absence of RO-3306, 78% of MN PCCs are in G1 and early-S phase, while only 22% are in mid-S, late-S, and G2 phase. In the presence of RO-3306 for 20 h, only 17% of MN PCCs are in G1 and early-S phase, while 40% are in mid-S, 26% in late-S, and 17% in G2. Following complete DNA replication, the anaphase lagging chromosomes entrapped in MN can proceed to G2 phase, without any apparent chromosome shattering. (Mean ± SEM based on four independent experiments; a total of 499 MN PCC spreads were analyzed; ** *p* ≤ 0.01, *** *p* ≤ 0.001).

In the second set of experiments, asynchronous micronucleated cells were generated by in vitro γ-irradiation with 4 Gy delivered to blood cultures at 17 h following their stimulation with PHA, i.e., at the G1/S phase border, as described in the section of Materials and Methods. Since the G1/S phase border is a highly radiosensitive stage, the rationale of this set of experiments was to induce an increased yield of chromosomal aberrations, resulting into an increased yield of MN, to further test our hypothesis. The percentages of cells in the five different categories of MN PCCs obtained under these experimental conditions, in the absence of RO-3306, are presented in Figure 7 (grey). The corresponding percentages obtained when micronucleated cells were treated with the RO-3306 agent for 20 h are also presented in Figure 7 (black). The results of this set of experiments confirm that, even under different experimental conditions for the generation of micronucleated cells, delaying the entry of main nuclei into mitosis by means of RO-3306 allows progression of DNA replication and progression of MN towards G2 phase.

### 2.3. The PCC Process Induces Shattering of Chromosomes in MN Still Replicating DNA When Main Nuclei Enter Mitosis in Asynchronous Micronucleated Cells Generated by γ-Irradiation of Mitotic CHO Cells

**Figure 8:**
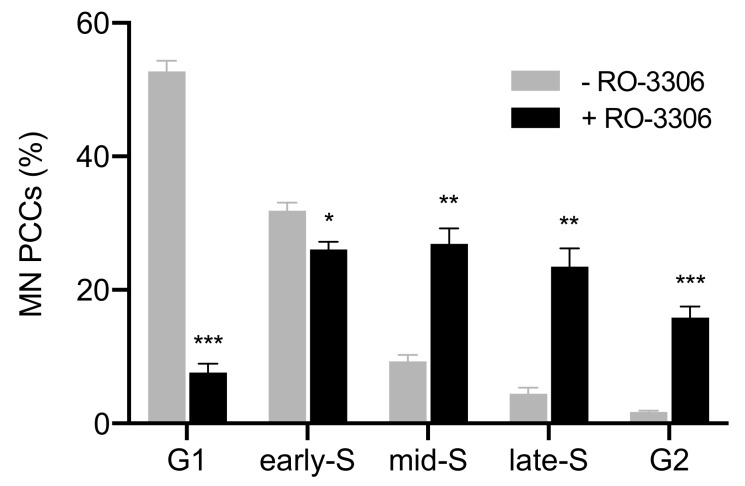
Cell cycle distribution of MN PCCs generated by irradiated mitotic Chinese hamster ovary (CHO) cells. Frequencies in five different cell cycle phase categories of MN PCCs scored in heterophasic micronucleated cells, generated by irradiation of CHO mitotic cells with 3 Gy γ-rays. In the absence of RO-3306, 85% of MN PCCs are in G1 and early-S phase, and only 15% are in mid-S, late-S, and G2 phase. In the presence of RO-3306 for 12 h, 33% are in G1 and early-S phase, while 27% of MN PCCs are in mid-S, 24% in late-S, and 16% in G2 phase. The presence of RO-3306 in micronucleated cells effectively delayed the main nuclei to proceed to mitosis, allowing time for the progression of DNA replication in chromosomes entrapped in MN. (Mean ± SEM based on four independent experiments; a total of 958 MN PCC spreads were analyzed; * *p* ≤ 0.05, ** *p* ≤ 0.01, *** *p* ≤ 0.001).

In the third set of experiments, asynchronous micronucleated cells were generated by in vitro γ-irradiation (3 Gy) of M phase CHO cells harvested via cell synchronization and selective detachment, as described in the section of Materials and Methods. The M phase is known to be extremely radiosensitive and the rationale of the experimental design was, again, to induce an increased yield of chromosomal aberrations, in order to obtain an increased yield of asynchronous micronucleated cells. The percentages of cells in the five different categories of MN PCCs under these experimental conditions in the absence of the RO-3306 agent are presented in Figure 8 (grey), while the black columns in Figure 8 present the corresponding results in the presence of the agent. The results confirm again that the presence of RO-3306 allowed time for the progression of DNA replication in the chromosomes entrapped in MN, alleviating the shattering effect.

### 2.4. The Presence of Asynchronous Mitosis in Multinucleated Cells Generated by Fusion of Exponentially Growing Cells Is an Important Determinant of the Shattering of Genetic Material in a Single Catastrophic Event

**Figure 9:**
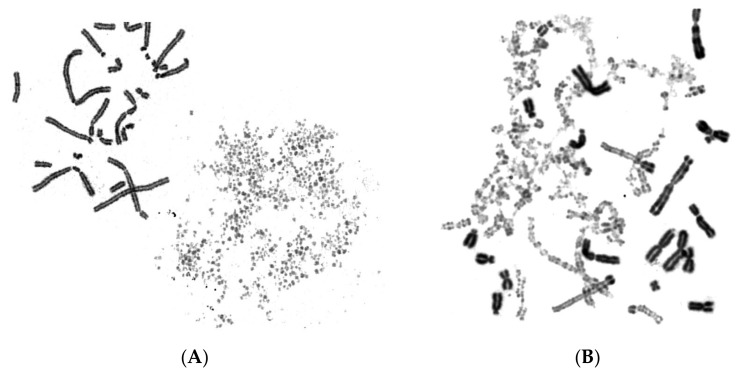
Early-S and late-S induced PCCs in multinucleated CHO cells. When main nuclei in heterophasic multinucleated cells generated by cell fusion procedures using exponentially growing CHO cells enter mitosis, the shattering and morphology of prematurely condensed chromosomes (PCCs) characterizes the stage in S phase of interphase nuclei. Based on the degree of completion of DNA replication, the induced PCCs in interphase nuclei can be classified as early-S (**A**) and late-S phase (**B**).

**Figure 10:**
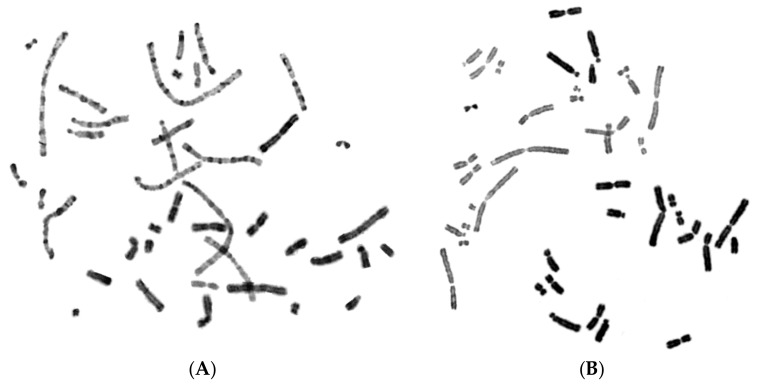
Early-G2 and late-G2 induced PCCs in multinucleated CHO cells. Based on the degree of completion of DNA replication in interphase nuclei in CHO multinucleated cells, the induced PCCs in interphase nuclei, upon entry of main nuclei into mitosis, can be classified as early-G2 with long lightly stained double chromatid chromosomes (**A**) or late-G2 with short lightly stained double chromatid chromosomes (**B**). The darkly stained condensed metaphase chromosomes belong to the main nuclei. Chromosome shattering in G2 phase PCCs was never observed.

To further test the proposed hypothesis, in the fourth set of experiments, asynchronous multinucleated cells were generated by the cell fusion procedure using exponentially growing CHO cells and the fusogen polyethylene glycol, as described in the section of Materials and Methods. The rationale of this set of experiments is based on the fact that, when a nucleus in asynchronous multinucleated cells is in S phase, while a neighboring nucleus proceeds to mitosis, the chromosomes in the S phase nucleus will be forced to condense prematurely and will shatter when the neighboring nucleus enters mitosis. According to the degree of completion of DNA replication, the chromosomes visualized in interphase nuclei can be classified as being in early-S or late-S, shown in Figure 9, and early-G2 or late-G2, shown in Figure 10.

**Figure 11:**
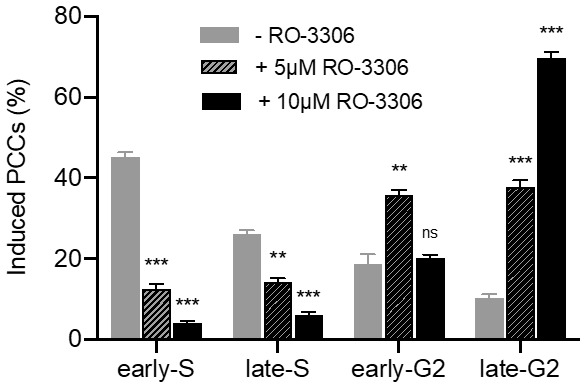
Cell cycle distribution of induced PCCs in multinucleated cells generated by fusion of asynchronous CHO cells. Frequencies in four different cell cycle phase categories of PCCs in asynchronous heterophasic multinucleated cells generated by cell fusion procedures using exponentially growing CHO cells. In the absence of RO-3306, 45% of PCCs observed are in early-S, 26% in late-S phase, 19% in early-G2, and 10% in late-G2; whereas in the presence of 5 μM RO-3306 for 12 h, only 12% were in early-S, 14% in late-S, 36% in early-G2, and 38% in late-G2. When 10 μM RO-3306 was used to inhibit CDK1 and delay more effectively the entrance of main nuclei into mitosis, 90% of heterophasic nuclei proceeded into early-G2 and late-G2, without any chromosome shattering, whereas only 10% of PCCs were left in early-S and late-S cell cycle phase, exhibiting chromosome shattering. The use of RO-3306 to synchronize heterophasic nuclei in multinucleated cells demonstrates that the extent of chromosome shattering is inversely related to the degree of synchronization achieved. (Mean ± SEM based on three independent experiments; a total of 1083 induced PCC spreads were analyzed; ns *p* > 0.05, ** *p* ≤ 0.01, *** *p* ≤ 0.001).

The percentages of PCCs obtained in the absence of RO-3306 (grey), or in the presence of 5 μM (black diagonal) or 10 μM (black) of the agent, are shown in Figure 11. The synchronization of heterophasic nuclei is more effective when 10 μM of the agent RO-3306 is applied.

These results demonstrate that chromosome shattering is induced in interphase nuclei by the dynamics of premature chromosome condensation in asynchronous multinucleated cells and depends on the stage of the nucleus in S phase. Indeed, the use of RO-3306 to synchronize the cell cycle phase of the nuclei of multinucleated cells shows that the extent of chromosome shattering is inversely related to the degree of synchronization achieved, which is more effective when 10 μM of the agent is used. In fact, as the nuclei complete DNA replication and proceed to G2 phase, there is no chromosome shattering when the main nucleus enters mitosis.

## 3. Discussion

Chromothripsis is a phenomenon characterized by chaotic localized rearrangements, all curiously restricted to one or a few chromosomes. Specifically, at the cytogenetic level, a simple model that fits the observed rearranged chromosomes, most likely entrapped in MN, involves a shattering of chromosomes into pieces with narrowly spaced breakpoints. This is initiated by a one-step cellular catastrophic event, followed by reassembly of the chromosomal segments in random order and orientation. As a result, some fragments are lost and others preserved into functioning highly derivative chromosomes, so that an alternating pattern of heterozygosity emerges [40]. Consequently, chromothripsis provides a mechanism for the rapid accumulation of hundreds of rearrangements in few cell divisions, in contrast to the traditional view of carcinogenesis as a gradual Darwinian process of progressive mutation accumulation. Yet, considering that chromosome shattering usually reflects induction of DSBs and DNA fragmentation [41,42], a number of questions have been raised: How is shattering of the genetic material initiated by a one-step cellular catastrophic event? Why is shattering confined to only one or a few chromosomes? How is the shattered DNA not lost but integrated into the genome? Which DNA repair mechanisms are involved? [21,24]. In addition, at which stage are DSBs generated in MN? Does the massive accumulation of DNA damage that leads to chromosome shattering and chromothripsis originate from MN disruption or aberrant DNA replication in MN before main nuclei enter mitosis? 

It has been previously proposed that the physical isolation of chromosomes in aberrant nuclear structures called MN, which are considered to be built with dysfunctional nuclear envelopes [27,43], might explain the localization of DNA lesions in chromothripsis [25]. However, the interesting question that still remains is why does DNA in MN get fragmented as the cell progresses to the next cycle? It has been shown that many processes taking place in the main nucleus are dysfunctional in the MN [27], including transcription and DNA replication [25,44]. Therefore, DNA damage can result as a direct consequence of aberrant DNA replication, potentially due to a reduced density of replication origins. DSBs could then be generated by stalled or slowed replication forks [45], but why forks are so fragile in mitosis is unclear. Furthermore, it has been reported that the ensuing loss of nucleocytoplasmic compartmentalization throughout interphase triggers micronucleus-specific DSBs, which were hypothesized to persist unrepaired into mitosis. These DSBs are expected to subsequently resolve into highly fragmented chromatin during mitotic entry [25,33,46], which reintegrates into daughter cell genomes after mitotic exit [13,25]. In addition, defects in repair inside MN have been reported to be associated with defects in the assembly of nuclear pore complexes on the MN envelope [35,47]. This may prevent recruitment of DNA replication or repair enzymes inside MN, producing DNA breakage [47]. 

Further work has shown that MN in cancer cells may undergo rupture of the MN envelope, which is associated with loss of compartmentalization and extensive DNA damage, and that this rupture represents an essential step for chromosome shattering to occur [27]. These findings led to the hypothesis that envelope disruption could expose DNA in the MN to cytoplasmic components, including endo and exonucleases that recognize collapsed replication forks or unrepaired DNA extruded from the MN, thereby generating pulverized chromosomal regions at mitotic entry [27]. Finally, DNA replication in MN was found to be asynchronous relative to the main nucleus [25,26]. Therefore, if the replicating DNA within the MN is exposed to “mitotic signals” when the main nucleus is in mitosis, the micronuclear envelope disassembles, while the residing chromosomes could potentially undergo PCC and pulverization, a phenomenon that was observed long ago [46,48,49]. Using different cytogenetic approaches, we previously provided experimental evidence supporting the above PCC model for chromosome shattering in MN as the mechanism underlying chromothripsis initiation [26]. Specifically, we reported that PCC induction in micronucleated cells exerts mechanical stress and shattering of chromosome segments still undergoing DNA replication in MN, when main nuclei enter mitosis. These results suggest that replication forks, which are sensitive to mechanical stress, may be directly damaged and collapse through premature chromatin condensation at the DNA replicating sites, causing chromosome shattering.

In the present study, we further assessed whether the dynamics of the PCC process studied earlier [26,37,39] represent an important determinant of the mechanistic origin of chromothripsis. The experimental design we used allowed for the examination of whether massive accumulation of DSBs, which underlie chromosome shattering and chromothripsis, originate necessarily through MN disruption or aberrant DNA replication in MN, before main nuclei enter mitosis. Through four different experimental setups and using asynchronous micronucleated or multinucleated cells, we demonstrated that a decisive parameter for the extent of chromosome shattering is the degree of cell cycle asynchrony between MN and main nuclei. Indeed, using asynchronous micronucleated cells generated by in vitro γ-irradiation of G0 human blood lymphocytes combined with the agent RO-3306, a thorough cytogenetic assessment was carried out at the time main nuclei proceeded to mitosis.

The induced MN PCCs were classified as G1, early-S, mid-S, late-S, or G2 phase, as shown in Figure 1 and Figure 2. Analysis of MN PCCs in asynchronous micronucleated cells was possible only if main nuclei were in M phase, as explained in Figure 3. The results presented in Figure 4 show that in the absence of RO-3306, 75% of MN PCCs are in G1 and early-S phase, and only 25% are of mid-S, late-S, and G2 phase. Whereas in the presence of RO-3306, 36% of MN PCCs are in mid-S, 32% in late-S, 21% in G2, and only 11% are now in G1 and early-S phase. As the DNA replication is progressing and MN proceed towards G2, the observed chromosome shattering decreases. In fact, the extent of chromosome shattering increases as the number of MN replication forks becomes larger at the entry of main nuclei into mitosis. Furthermore, we show that, if given sufficient time, chromosomes entrapped in MN can undergo chromatid disjunction (Figure 2A) and complete DNA replication (Figure 2B), without any impairment in their duplication or shattering, as it is demonstrated for chromosomes 1, 4 and a fragment (Figure 5A), and for chromosomes 2 and 10 (Figure 5B). If given sufficient time, radiation-induced aberrant chromosomes entrapped in MN can also replicate their DNA, without any apparent chromosome shattering, as shown in Figure 6. 

If disruption, aberrant DNA replication, or accumulation of MN-specific DSBs that can persist unrepaired into G2/M phase had taken place in MN before the entry of main nuclei into mitosis, then the duplication of chromosomal material residing in MN would have been markedly impaired and chromosome shattering would be expected to be present in (at least) some of the sites of MN G2-PCCs. However, following a thorough interphase PCC assessment upon the entry of main nuclei into mitosis, no such shattering was observed in any of the MN G2-PCCs analyzed. Therefore, under the experimental conditions used in this work, the presence of unrepaired DSBs in MN could not be confirmed. Yet, the results obtained demonstrate that the PCC dynamics during asynchronous mitosis in micronucleated or multinucleated cells are an important determinant of chromosome shattering in a single catastrophic event, suggesting that they may underlie the mechanistic origin of chromothripsis.

Similar results were obtained from the second set of experiments using micronucleated cells generated by γ-irradiation of PHA-stimulated lymphocytes at the highly radiosensitive G1/S border to induce an increased yield of chromosomal aberrations and, thus, an increased yield of MN. Figure 7 shows that in the absence of RO-3306, 78% of MN PCCs are in G1 and early-S phase, and only 22% are in mid-S, late-S, and G2 phase. Whereas in the presence of RO-3306, 40% of MN PCCs are in mid-S, 26% in late-S, 17% in G2, and only 17% are now in G1 and early-S phase. In the third set of experiments, an increased yield of asynchronous micronucleated cells was generated by in vitro γ-irradiation of M phase CHO cells. Figure 8 shows that in the absence of RO-3306, 85% of MN PCCs are in G1 and early-S phase, and only 15% are in mid-S, late-S, and G2 phase. Whereas in the presence of RO-3306, 27% of MN PCCs are in mid-S, 24% in late-S, 16% in G2, and 33% are now in G1 and early-S phase. The presence of the RO-3306 agent in micronucleated cells effectively delayed the main nuclei to proceed to mitosis, thus allowing time for the progression of DNA replication in the chromosomes entrapped in MN. These results confirm that, when the RO-3306 agent is used in micronucleated cells generated under different experimental conditions in order to delay entry of main nuclei into mitosis, the DNA replication in MN continues, showing less chromosome shattering as they proceed in the cell cycle and no shattering upon their entry in G2 phase.

In the fourth set of experiments, the proposed hypothesis was tested in asynchronous heterophasic multinucleated cells generated by cell fusion procedures using exponentially growing CHO cells. The interphase nuclei in these multinucleated cells show different PCC morphologies and shattering upon the entry of main nuclei into mitosis, which are characteristic of their stage in the cell cycle (Figure 9 and Figure 10). As shown in Figure 11, in the absence of RO-3306, 45% of PCCs observed are in early-S, 26% in late-S, 19% in early-G2, and 10% in late-G2. Whereas in the presence of 5 μM RO-3306, only 12% are in early-S, 14% in late-S, 36% in early-G2, and 38% in late-G2. The use of 10 μM RO-3306 to inhibit CDK1 and delay more effectively the entrance of main nuclei into mitosis, gave the best cell cycle synchronization between heterophasic nuclei. After releasing cells from RO-3306, thus allowing the main nuclei to enter mitosis, 90% of heterophasic nuclei proceeded into early-G2 and late-G2, without any chromosome shattering, whereas only 10% of PCCs were still in early-S and late-S, as shown also in Figure 11. These results demonstrate that chromosome shattering is induced by the dynamics of premature chromosome condensation in asynchronous multinucleated cells and depends on the stage of the nucleus in S phase when main nuclei enter mitosis. Indeed, the use of RO-3306 to synchronize heterophasic nuclei in multinucleated cells shows that the extent of chromosome shattering is inversely related to the degree of synchronization achieved. 

Altogether, the results obtained demonstrate that the PCC dynamics are an important determinant of the mechanistic origin of the chromosome shattering that is a hallmark of chromothripsis. They also suggest that the massive accumulation of DSBs that underlie chromothripsis may not necessarily originate through MN disruption or aberrant DNA replication in MN, before main nuclei enter mitosis. Alternatively, when main nuclei enter mitosis, the PCC dynamics, provoked by chromatin condensation, can exert a mechanical stress on the DNA replication sites of chromosomes entrapped in MN, or interphase nuclei in multinucleated cells, still undergoing DNA replication. As a result, the DNA replication forks in MN may collapse and eventually convert into DSBs, causing thus the observed chromosome shattering in a single catastrophic event. The extent of chromosome shattering decreases as the MN proceed towards G2 and the number of replication forks becomes smaller at the entry of main nuclei into mitosis. 

Can the mechanical stress exerted by premature chromatin condensation on DNA replicating sites cause collapse of replication forks? In agreement with our findings, Falk et al. very recently provided experimental evidence supporting that chromatin architecture changes can induce replication fork collapse. Specifically, the authors, using DNA replicating cells, demonstrated that chromatin condensation, provoked by the freeze-thaw process, causes replication fork collapse, potentially leading to DSBs, which represent an important source of genome instability [50]. Yet, experimental evidence for a similar impact of interphase chromatin architecture changes on radiation-induced DNA damage repairing sites was already recognized and reported in our earlier work [37,38,39]. 

The mechanism proposed in the present work may help to deepen our understanding of most of the criteria for chromothripsis, including the important one related to why chromothriptic genome profiles exhibit clustering of DNA breaks. Such clustering is defined by specific chromosomal regions having multiple breaks in close proximity, surrounded by large sections of intact chromosomal sequence. Considering the pattern of DNA replication and distribution of replication sites in a chromosome segment progressing towards G2, the chromothriptic genome profiles obtained can be justified. Indeed, only the segments of the genome still undergoing replication when main nuclei enter mitosis are affected by the dynamics of the induced PCC process. One envisions that the affected genome can be surrounded by large sections that have completed DNA replication, thus demonstrating intact chromosomal sequences. 

## 4. Materials and Methods

### 4.1. Lymphocyte Whole Blood Cultures, Production of Micronucleated Cells, and Irradiation Conditions

Peripheral blood samples in heparinized tubes were obtained from healthy donors and used after their informed consent, according to our institutional ethics procedures. The ethical approval was granted on the 26 February 2016, taking into consideration the Hellenic Law (Ν. 2472/97 and 886/Β’ 20/12/84) and the European Community Directives (Directive 95/46/EC). Irradiation of whole blood samples was carried out in vitro using a Co-60 Gamma Cell 220 irradiator (Atomic Energy of Canada Ltd., Ottawa, OT, Canada) at room temperature and at a dose rate of 20 cGy/min. Different irradiation times were applied in order to administer to the whole blood samples doses. 

For the first set of experiments, micronucleated cells were generated using irradiation of blood with 4 Gy to induce chromosomal aberrations and formation of micronuclei (MN). Following radiation exposure, cultures were set up for 72 h at 37 °C, by adding for each 0.5 mL of whole blood, 5 mL of RPMI-1640 medium (Gibco) supplemented with 10% fetal bovine serum (FBS), 1% Phytohaemagglutinin (PHA), and 1% glutamine and antibiotics (penicillin: 10,000 U/mL; streptomycin: 10,000 μg/mL (Biochrom GmbH, Berlin, Germany)). For the PCC analysis of interphase MN in the absence of the CDK1 inhibitor RO-3306 (Tocris, Abingdon, UK), Colcemid at a final concentration of 0.1 μg/mL (Gibco) was added to the blood cultures for 5 h before their harvest at 72 h for chromosome preparation. For the categorization of interphase MN PCCs after delaying the entrance of main nuclei into mitosis by means of RO-3306, blood cultures were centrifuged at 72 h culture time, resuspended in fresh culture medium with 10 μM final concentration of RO-3306 (in DMSO), and the cells were further incubated at 37 °C for 20 h. At that time, cultures were centrifuged and washed twice to remove RO-3306, and cells resuspended in fresh culture medium plus Colcemid and cultured for additional 5 h, before they were harvested for chromosome preparation. Chromosome spreads were prepared by standard cytogenetic procedures and air-dried slides were stained in 3% Giemsa solution for interphase analysis of MN PCCs induced at the entry of main nuclei into mitosis. The corresponding data presented in Figure 4 are mean values ± SEM based on two independent experiments. A total of 457 MN PCC spreads were analyzed. Statistical significance was determined by means of five unpaired *t*-tests, corrected for multiple comparisons using the Holm-Sidak method with alpha = 0.05. Each row was analyzed individually, without assuming a consistent SD. Asterisks indicate statistical significance of the difference between untreated and treated samples; * *p* ≤ 0.05, ** *p* ≤ 0.01, *** *p* ≤ 0.001.

For the second set of experiments, the same procedures were followed, except that the dose of 4 Gy was delivered to blood cultures at 17 h following their stimulation with PHA, i.e., as they were approaching the highly radiosensitive G1/S border. The corresponding data presented in Figure 7 are mean values ± SEM based on four independent experiments. A total of 499 MN PCC spreads were analyzed. The statistical analysis was performed following the same method as in the first set of experiments.

### 4.2. Micronucleated Cells Generated by Irradiation of Mitotic CHO Cells

Chinese hamster ovary cells (CHO-K1) were grown in McCoy’s 5A (Biochrom) culture medium supplemented with 5% FBS, 1% l-glutamine, and 1% antibiotics (Penicillin, Streptomycin), incubated at 37 °C in a humidified atmosphere with 5% CO_2_. CHO cultures were maintained as exponentially growing monolayer cultures in 75 cm^2^ plastic flasks at an initial density of 4 × 10^5^ cells/flask. For the third set of experiments, mitotic CHO cells were harvested via cell synchronization and selective detachment. The cells in a flask were routinely allowed to grow until confluence and, subsequently, subcultured into three new 75 cm^2^ plastic flasks. Following a 24–30 h incubation at 37 °C, Colcemid (Gibco) at a final concentration of 0.1 μg/mL was added to CHO cultures for 4 h and the accumulated mitotic cells were harvested by selective detachment. To generate CHO micronucleated cells, CHO mitotics were irradiated to 3 Gy and cultured at 37 °C for 24 h after their release from the colcemid block. 

For the PCC analysis of interphase MN in CHO micronucleated cells in the absence of RO-3306 CDK1 inhibitor, Colcemid at a final concentration of 0.1 μg/mL (Gibco) was added to the cultures 5 h before their harvest at 24 h for chromosome preparation. For the categorization of interphase MN PCCs after delaying the entrance of the CHO main nuclei into mitosis by means of the RO-3306 inhibitor, cultures were centrifuged at 24 h culture time, resuspended in fresh culture medium with 10 μM RO-3306 final concentration, and the cells were further incubated at 37 °C for 12 h. At that time, cultures were centrifuged and washed twice to remove RO-3306, and cells resuspended in fresh culture medium plus Colcemid and cultured for additional 5 h, before they were harvested for chromosome preparation. Four independent experiments were carried out and the corresponding data presented in Figure 8 are mean values ± SEM. A total of 958 MN PCC spreads were analyzed. The statistical analysis was performed following the same method as in the first set of experiments.

### 4.3. Heterophasic Multinucleated Cells Produced by Cell Fusion of Exponentially Growing CHO Cells

For the fourth set of experiments, cell fusion of exponentially growing CHO cells to generate heterophasic multinucleated cells, using 45% polyethylene glycol (PEG, p5402 Sigma–Aldrich, St. Louis, MO, USA) in serum-free RPMI-1640 medium with HEPES, was performed essentially as described previously [51]. Briefly, exponentially growing CHO cells harvested by trypsinization in 15 mL round-bottom culture tubes were used. After centrifugation at 1200 rpm for 8 min, the supernatant was discarded without disturbing the cell pellet, and cells were resuspended in 10 mL serum-free RPMI-1640 medium with HEPES. Following centrifugation at 1000 rpm for 8 min, the tubes were kept inverted in a test tube rack on a paper towel to drain the pellet from excess liquid. While holding each tube in an inverted position, 0.15 mL of PEG was injected forcefully against the cell pellet using a micropipette and, immediately after, the tube was turned in an upright position and held for about 1 min. Subsequently, 1.5 mL of PBS was slowly added to each tube with gentle shaking and the cell suspension was centrifuged at 1000 rpm for 8 min. The supernatant was discarded, and the cell pellet was suspended gently in 1 mL RPMI-1640 complete growth medium with HEPES containing 1% PHA and 0.1 μg/mL Colcemid. The tubes were then incubated for 15 min at 37 °C for the cell fusion to take place. Immediately after, fused cells were plated in petri dishes with 10 mL of McCoy’s 5A complete culture medium and cultured for 20 h at 37 °C so that the asynchronous heterophasic multinucleated cells could be attached and proceed to the cell cycle. Upon the entry of main nuclei into mitosis, PCCs are induced and have the characteristic appearance of pulverized chromosomes for S phase cells, but without any chromosome shattering for G1 or G2-PCCs. 

For the PCC analysis of interphase CHO heterophasic nuclei in the absence of RO-3306 CDK1 inhibitor, Colcemid at a final concentration of 0.1 μg/mL (Gibco) and fresh culture medium were added to the fused cells for 5 h before their harvest for chromosome preparation. For the categorization of interphase PCCs after delaying the entrance of the main CHO nuclei into mitosis by means of the RO-3306 inhibitor, following the 20 h culture of hybrid cells at 37 °C, they were further incubated at 37 °C for 12 h in fresh culture medium containing 10 μM final concentration of RO-3306. At that time, culture dishes were washed twice to remove RO-3306, and cultured in fresh medium plus Colcemid for 5 h, before they were harvested for chromosome preparation by selective detachment. Cells were then treated with hypotonic KCl (0.075 M) and fixed with two changes of methanol:glacial acetic acid (v/v 3:1). The chromosome spreads were prepared by the standard air-drying technique and slides were stained using 3% Giemsa in Sorensen buffer solution for PCC analysis. Three independent experiments were carried out and the corresponding data presented in Figure 11 are mean values ± SEM, obtained by cytogenetic assessment of interphase nuclei in hybrid cells when the main CHO nuclei entered mitosis. A total of 1083 induced PCC spreads were analyzed. Statistical significance was determined by means of four unpaired *t*-tests for each condition, corrected for multiple comparisons using the Holm-Sidak method with alpha = 0.05. Each row was analyzed individually, without assuming a consistent SD. Asterisks indicate statistical significance of the difference between untreated and treated samples; ns *p* > 0.05, * *p* ≤ 0.05, ** *p* ≤ 0.01, *** *p* ≤ 0.001. 

## 5. Conclusions

The results obtained provide direct experimental evidence indicating that the occurrence of asynchronous mitosis in heterophasic micronucleated or multinucleated cells is an important determinant of the shattering of genetic material in a single catastrophic event, which is a hallmark of chromothripsis. Chromosome shattering and genomic rearrangements appear to depend on the number of replication forks and the degree of completion of DNA replication, in MN or in heterophasic nuclei in multinucleated cells, when main nuclei enter mitosis. Consistent with our recent work [26], the results here support a simple model to explain how shattering of the genetic material can be initiated by a one-step cellular catastrophic event, and why it can be confined to one or a few chromosomes, or a chromosome arm. This model is an alternative to the one that considers nuclear envelope rupture or aberrant DNA replication in MN resulting in DSBs that must persist unrepaired into the next mitosis.

The mechanical stress exerted by chromatin condensation on sites still undergoing normal DNA replication in MN when main nuclei enter mitosis has, in itself, the potential to induce the collapse of replication forks that may develop into DSBs and cause chromosome shattering. Moreover, the extent of chromosome shattering depends on the magnitude of chromosome segments with late DNA replicating sites when main nuclei enter mitosis. Although shattering can possibly affect multiple chromosomes as well, leading to random fusion of their fragments, one envisions that only cells with one or a few rearranged chromosomal regions could survive and be detected in real cancers, probably determined by a selective advantage. When cells are affected by severe DNA damage, they undergo apoptosis, and this may happen in most cases when chromothripsis affects multiple chromosomes. Exceptionally, however, chromothripsis can cause disruption of tumor suppressor genes and activation of oncogenes, leading to cell survival.

Altogether, our results demonstrate that the dynamics of the PCC process arising during asynchronous mitosis in micronucleated or multinucleated cells, known to take place also in vivo [52], are an important determinant of chromosome shattering in a single catastrophic event, suggesting that they may underlie the mechanistic origin of chromothripsis. Among the several non-mutually exclusive cellular mechanisms that have been proposed, the present work supports the PCC hypothesis in MN. Nevertheless, additional studies are needed to provide definitive insights into this striking phenomenon and, subsequently, into its role in cancer initiation and progression. Deciphering chromothripsis may also be crucial for the development of strategies to interfere with the underlying processes in a therapeutic setup.
